# Japanese Planocerid Flatworms: Difference in Composition of Tetrodotoxin and Its Analogs and the Effects of Ingestion by Toxin-Bearing Fishes in the Ryukyu Islands, Japan

**DOI:** 10.1007/s10126-024-10312-0

**Published:** 2024-04-17

**Authors:** Hiroyuki Ueda, Masaaki Ito, Ryo Yonezawa, Kentaro Hayashi, Taiga Tomonou, Maho Kashitani, Hikaru Oyama, Kyoko Shirai, Rei Suo, Kazutoshi Yoshitake, Shigeharu Kinoshita, Shuichi Asakawa, Shiro Itoi

**Affiliations:** 1 Department of Marine Science and Resources, Nihon University, Fujisawa, Kanagawa 252-0880 Japan; 2 Department of Aquatic Bioscience, Graduate School of Agricultural and Life Sciences, The University of Tokyo, Tokyo, 113-8657 Japan; 3 Present Address: Biomedical Research Institute, National Institute of Advanced Industrial Science and Technology (AIST), 1-1-1 Higashi, Tsukuba, Ibaraki 305-8566 Japan

**Keywords:** Tetrodotoxin (TTX), TTX analogs, Planocerid, Pufferfish, Toxic flatworm

## Abstract

Tetrodotoxin (TTX), known as pufferfish toxin, is a potent neurotoxin blocking sodium channels in muscle and nerve tissues. TTX has been detected in various taxa other than pufferfish, including marine polyclad flatworms, suggesting that pufferfish toxin accumulates in fish bodies via food webs. The composition of TTX and its analogs in the flatworm *Planocera multitentaculata* was identical to those in wild grass puffer *Takifugu alboplumbeus*. Previously, *Planocera* sp. from Okinawa Island, Japan, were reported to possess high level of TTX, but no information was available on TTX analogs in this species. Here we identified TTX and analogs in the planocerid flatworm using high-resolution liquid chromatography–mass spectrometry, and compared the composition of TTX and analogs with those of another toxic and non-toxic planocerid species. We show that the composition of TTX and several analogs, such as 5,6,11-trideoxyTTX, dideoxyTTXs, deoxyTTXs, and 11-norTTX-6(*S*)-ol, of *Planocera* sp. was identical to those of toxic species, but not to its non-toxic counterpart. The difference in the toxin composition was reflected in the phylogenetic relationship based on the mitochondrial genome sequence. A toxification experiment using predatory fish and egg plates of *P. multitentaculata* demonstrated that the composition of TTX and analogs in wild *T. alboplumbeus* juveniles was reproduced in artificially toxified pufferfish. Additionally, feeding on the flatworm egg plates enhanced the signal intensities of all TTX compounds in *Chelonodon patoca* and that of deoxyTTXs in *Yongeichthys criniger*.

**Supplementary Information:**

The online version contains supplementary material available at 10.1007/s10126-024-10312-0.

## Introduction

Tetrodotoxin (TTX), known as pufferfish toxin, is one of the most potent natural toxins, inhibiting neurotransmission by blocking sodium channels in muscle and nerve tissues (Narahashi 2001). TTX is found in a wide variety of marine organisms that are prey of pufferfish, such as gastropods, crustaceans, ribbonworms, and flatworms (Noguchi et al. 2006; Noguchi and Arakawa 2008; Bane et al. 2014; Magarlamov et al. 2017; Katikou et al. 2022). In addition, non-toxic pufferfish can be produced by rearing them on TTX-free feed, and these non-toxic pufferfish rapidly toxify when fed TTX-containing feed, suggesting that TTX in wild pufferfish tissues is derived from prey organisms (Matsui et al. 1981, 1982; Honda et al. 2005; Noguchi et al. 2006; Itoi et al. 2015). However, although the biosynthetic mechanism of TTX has been inferred from the discovery of various TTX analogs (Kudo et al. 2014a, b, 2016, 2020, 2021; Ueyama et al. 2018; Kudo and Yotsu-Yamashita 2019), there is no information on the gene cluster involved in this process.

The search for missing links among TTX-bearing organisms, especially relationships relative to pufferfish, leads to TTX-bearing flatworms. For example, it has been shown that the toxification of the sea slug *Pleurobranchaea maculata*, the causative organism of dog poisoning in New Zealand, resulted from feeding on the flatworm *Stylochoplana* sp. (Salvitti et al. 2015). It has been also shown that grass puffer *Takifugu alboplumbeus* ingested TTX by feeding on the flatworm *Planocera multitentaculata* throughout its life history (Itoi et al. 2018; Okabe et al. 2019), and that juveniles of milk-spotted puffer *Chelonodon patoca* and toxic goby *Yongeichthys criniger* ingested TTX by feeding on larvae of *P. multitentaculata* or related species (Itoi et al. 2020a; Ito et al. 2022). Furthermore, planocerid flatworm larvae have been reported to be involved in the toxification of bivalves with TTX (Okabe et al. 2021), which has become a worldwide problem in recent years (EFSA Contam Panel et al. 2017; Biessy et al. 2019; Antonelli et al. 2022; Yasukawa et al. 2023).

No TTX-free individuals were detected in the flatworm *P. multitentaculata*, and that the amount of TTX in the body increased with body size (Yamada et al. 2017; Itoi et al. 2020b). This flatworm is recognized as an important key organism in the investigation of TTX origin. Recently, it has been reported that *P. multitentaculata* possess a variety of TTX analogs, including dideoxyTTXs, deoxyTTXs, and 11-norTTX-(*S*)-ol, in addition to large amounts of TTX and 5,6,11-trideoxyTTX (Suo et al. 2022). Although the internal localization of these TTX analogs in the flatworm changes during growth (Oyama et al. 2022), there is no difference in the composition of TTX analogs between samples collected in different sites (Suo et al. 2024), indicating that the flatworm might possess biosynthetic processes of TTX in the body. On the other hand, there is no information on TTX analogs in planocerid flatworms, with the exception of the planocerid in Guam (Ritson-Williams et al. 2006) and *P. multitentaculata* as mentioned (Suo et al. 2022, 2024).

The 5,6,11-trideoxyTTX has been reported to be dominantly high in a variety of organisms (Rambla-Alegre et al. 2017; Suo et al. 2021, 2022, 2024; Ito et al. 2022, 2023; Vlasenko and Magarlamov 2020, 2022). Recently, TTX/5,6,11-trideoxyTTX ratios of pufferfish, including *C. patoca*, collected throughout the Japanese Archipelago, ranged from 2 to 5 regardless of species or region. Meanwhile, the ratios within toxic goby *Y. criniger* collected in Okinawa and Ishigaki Island, including individuals collected at the same sites as *C. patoca*, were found to differ markedly depending on the date/location of sampling (Ito et al. 2022, 2023). This suggests not only that *C. patoca* and *Y. criniger* feed on different organisms but also that the accumulation mechanism of TTX analogs differs between them and that the composition of TTX analogs in the bodies of *Y. criniger* is affected by the composition of TTX analogs in their prey.

In this study, we sought to accumulate the basic knowledge required to elucidate the source of TTX and its analogs in pufferfish and toxic goby. Additionally, we attempted to reveal the composition of TTX and its analogs in four species of Japanese planocerid, and to clarify the factors affecting changes in the composition of TTX and analogs in pufferfish and toxic goby after feeding on the egg plates of *P. multitentaculata*.

## Materials and Methods

### Flatworms

**Fig. 1:**
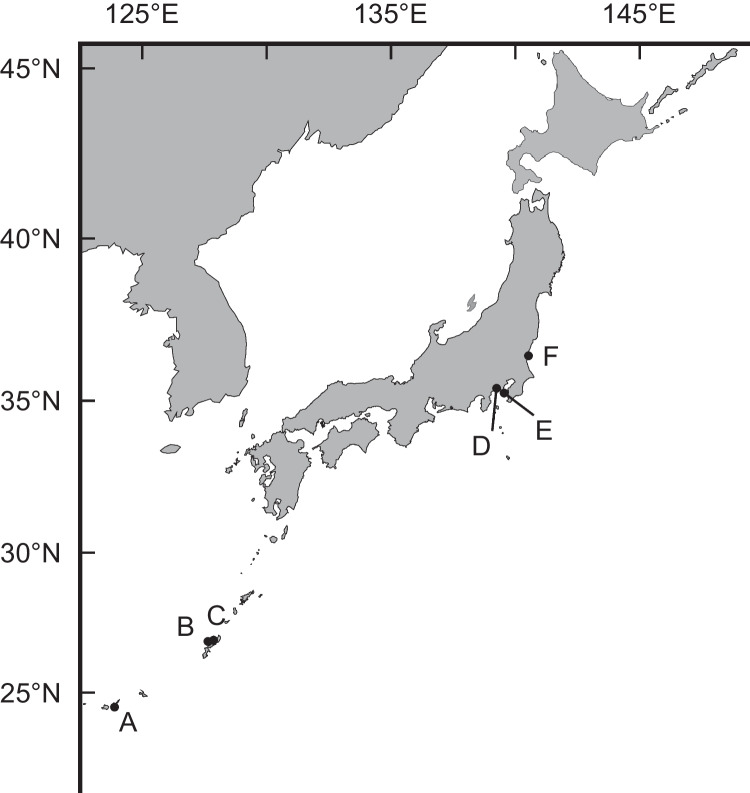
Sampling localities for planocerid flatworms used in this study

**Fig. 2:**
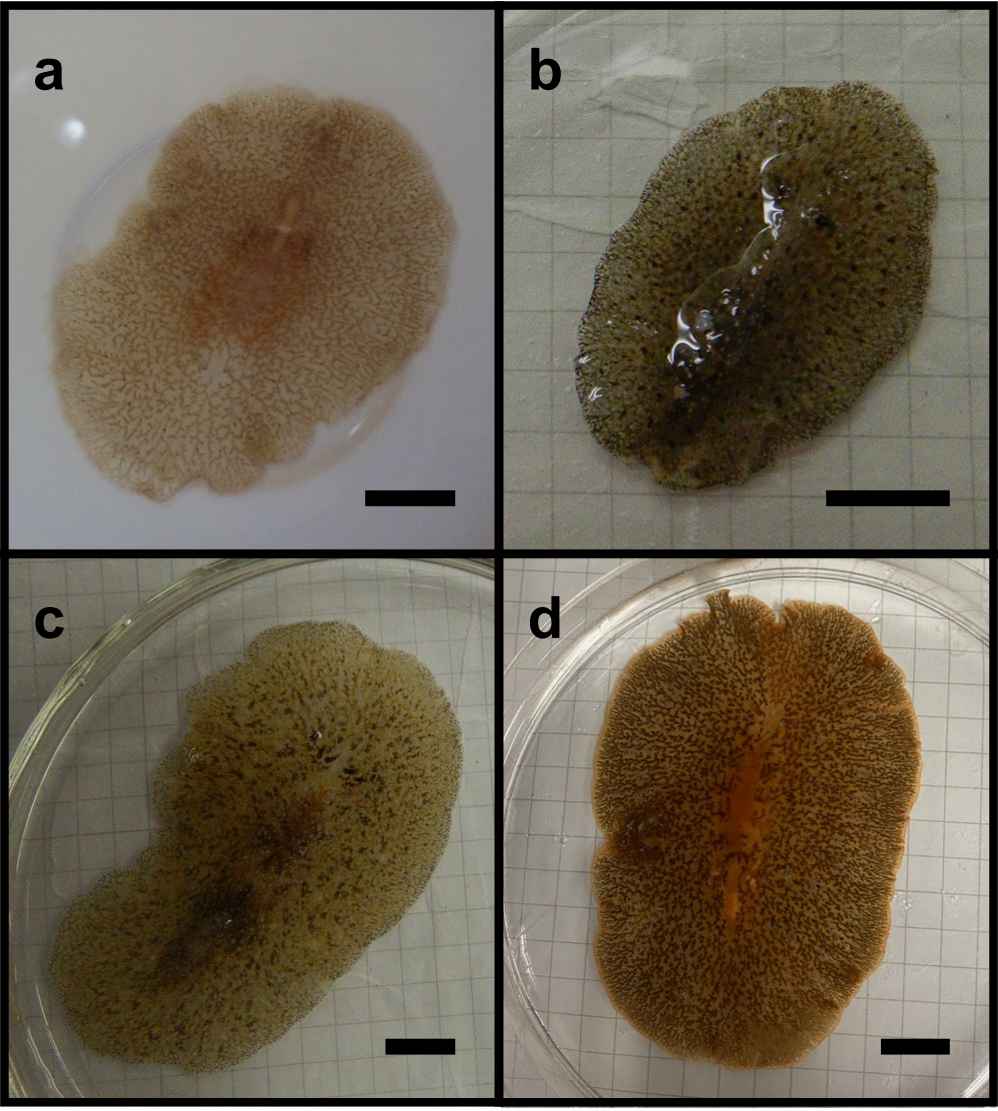
External morphology of planocerid flatworms used in this study. **a** *Planocera* sp.; **b** *Planocera reticulata*; **c** *Planocera multitentaculata*; **d** *Planocera pellucida*. Bars represent 10 mm

Four species of planocerid flatworms were collected from various locations in the Japanese Islands. *Planocera* sp. (Fig. 2a) were collected in August, 2017, under stones at a depth of 10 m off Nago, Okinawa Island in the Ryukyu Islands, Japan (“B” in Fig. 1; Ueda et al. 2018). *Planocera reticulata* (Fig. 2b) and *Planocera pellucida* (Fig. 2d) were collected in May, 2023, and May, 2019, respectively, under stones in coastal waters around Miura Peninsula, Kanagawa, Japan (“E” in Fig. 1), and *Planocera multitentaculata* (Fig. 2c) were collected in June, 2020, at Ibaraki, Japan (“F” in Fig. 1). These samples were stored at – 30 °C until use. Several specimens of *P. multitentaculata* were also collected in April, 2022, around Miura Peninsula, Kanagawa, Japan, and reared in a 60-L tank to collect egg plates for use in toxification experiment.

### Fish Juveniles

TTX-bearing juvenile fish (analyzed as wild individuals) were collected from various locations in the Ryukyu Islands, Japan as follows: juveniles of *Chelonodon patoca* (mean body weight 1.02 ± 0.44 g, *n* = 10) and *Yongeichthys criniger* (mean body weight 0.65 ± 0.26 g, *n* = 13) were collected on Ishigaki Island (“A” in Fig. 1) in June, 2022, whereas *Takifugu alboplumbeus* (mean body weight 0.84 ± 0.23 g, *n* = 10) were collected from Okinawa Island (“C” in Fig. 1) in May, 2022. These samples were stored at – 30 °C until analysis.

Juvenile fish used in the toxification experiments were prepared as follows: non-toxic juveniles of *T. alboplumbeus* (mean body weight 0.16 ± 0.06 g, *n* = 11) were raised using commercial feed pellets after artificial fertilization at Enoshima, Kanagawa, Japan (“D” in Fig. 1) in June, 2022, whereas juveniles of *C. patoca* (mean body weight 0.47 ± 0.13 g, *n* = 9) and *Y. criniger* (mean body weight 0.37 ± 0.18 g, *n* = 10) were collected on Ishigaki Island (“A” in Fig. 1) in June, 2022, and reared in laboratory tanks on non-toxic feed for 1 month. Non-toxic juveniles of *T. alboplumbeus* used in the intramuscular administration of TTX (mean body weight 1.16 ± 0.06 g, *n* = 3) or 5,6,11-trideoxyTTX (mean body weight 1.59 ± 0.15 g, *n* = 3) were artificially raised in 2023 in a similar procedure to that used for the toxification experiments.

### Toxification Experiment

To reproduce the composition of TTX and analogs in predators feeding on TTX-bearing flatworms, we conducted toxification experiments in which *T. alboplumbeus*, *C. patoca*, and *Y. criniger* were fed egg plates of *P. multitentaculata*, under the following conditions. Juvenile fish were housed individually in mesh-partitioned cages (70 mm length × 110 mm width × 70 mm depth; up to 12 compartments) within 15-L tanks under aeration. Fish were fed a flatworm egg plate (approx. 0.08 g) containing approx. 190 μg TTX and 180 μg 5,6,11-trideoxyTTX for 1 day (Ito et al. 2023). After the treatment, individuals of all three fish species were reared for 1 week on non-toxic commercial feed pellets, allowing for excretion of toxic flatworm egg plates. The composition of TTX and analogs in these juvenile fish was then measured by liquid chromatography–mass spectrometry (LC–MS) analysis (Suo et al. 2022).

### Intramuscular Administration

TTX and 5,6,11-trideoxyTTX were dissolved in 0.1% acetic acid, and fish were intramuscularly injected with TTX or 5,6,11-trideoxyTTX at a dose of 6 μg/individuals. TTX for use in calibration solution for quantification of TTX was purchased from FUJIFILM Wako Pure Chemicals (purity ≥ 90%), while 5,6,11-trideoxyTTX was purified in accordance with Miyazaki et al. (2024). The fish injected with 0.1 mL of acetic acid were used as control group. After the treatment, all individuals were reared for 1 week on non-toxic commercial feed pellets. The composition of TTX and analogs in these fish was then measured LC–MS analysis (Suo et al. 2022).

### Extraction of TTX and Its Analogs

Extracts from planocerid flatworms and juvenile fish containing TTX and its analogs were prepared in accordance with Suo et al. (2022). Flatworm tissue was homogenized in 0.01 M acetic acid followed by heating for 10 min in boiling water. After centrifugation at 12,000 × g for 5 min at 4 °C, the supernatant was recovered and filtered through a membrane of pore size 0.45 μm. The filtrate was adjusted to pH 6.0 with acetic acid and loaded onto an activated charcoal column equilibrated with water. After washing the column with water, the TTX and analogs were eluted with acetic acid/ethanol/water (2:50:49, v/v), and the recovered solution was concentrated and lyophilized. The residue was redissolved in an acid solution adjusted to pH 6.0, and loaded onto the Bio-Gel P-2 column equilibrated with water. After washing the column with water, the compounds were eluted with 0.03 M acetic acid, and the fractions containing TTX and analogs were combined and concentrated for LC–MS analysis.

### LC–MS Analysis

The prepared extracts from flatworms and juvenile fish were appropriately diluted and subjected to high-resolution LC–MS analysis according to the method of Suo et al. (2022). LC–MS was carried out on a Shimadzu LC-20AD solvent delivery system connecting to a SCIEX X500R Q-TOF mass spectrometer with an ESI source. The interface parameters for optimized conditions were set as follows: curtain gas, 45 psi; ion spray voltage, 5500 V; temperature, 700 °C; ion source gas 1, 70 psi; ion source gas 2, 60 psi; collision gas, 7 psi. The TTX standards and the flatworm sample extracts were analyzed by LC–MS using a TSKgel Amide-80 column (2.0 mm × 150 mm, 5 μm) with 16 mM ammonium formate in water/acetonitrile/formic acid (30:70:0.002, v/v) at a flow rate of 0.2 mL/min at 28 °C (Jang et al. 2010). The [M + H]^+^ ions were measured using the extracted-ion chromatography XIC (± 0.02) corresponding ions for 5,6,11-trideoxyTTX at *m*/*z* 272.1241, dideoxyTTXs at *m*/*z* 288.1190, 11-norTTX-6(*S*)-ol at *m*/*z* 290.0983, deoxyTTXs at *m*/*z* 304.1139, and TTX and 4-epiTTX at *m*/*z* 320.1088. TTX for use in calibration solution for quantification of TTX was purchased from FUJIFILM Wako Pure Chemicals (purity ≥ 90%). Chemically synthesized 5,6,11-trideoxyTTX (Adachi et al. 2014) was provided as an authentic preparation. The standard calibration curve was created using 1–100 ng/mL of standards (TTX and 5,6,11-trideoxyTTX) which showed good linearity and precision (TTX: *y* = 9952.5*x*, *R*^2^ = 0.99; 5,6,11-trideoxyTTX: *y* = 5898.2*x*, *R*^2^ = 0.97). The limit of detection (LOD) was determined based on signal to noise ratio (S/N: 3). LODs of TTX and 5,6,11-trideoxyTTX were 0.2 and 0.8 ng/mL, respectively.

### DNA Extraction, Sequencing, and Phylogenetic Analysis

Total genomic DNA was extracted from tissue using QIAamp FFPE Tissue Kit (Qiagen, Hilden, Germany) for two individuals of *Planocera* sp., DNeasy Blood & Tissue Kit (Qiagen) for three individuals of *P. pellucida*, and NucleoBond HMW DNA (Macherey–Nagel, Düren, Germany) for one individual of *P. pellucida*. Libraries were prepared using MGIEasy FS DNA Library Prep Set (MGI, Shenzhen, China) for two individuals of *Planocera* sp. and one individual of *P. pellucida*, and Nextera DNA Flex library Prep kit (Illumina, San Diego, CA, USA) for three individuals of *P. pellucida*. Next-generation sequencing analysis was performed using DNBSEQ-T7 (MGI) for two individuals of *Planocera* sp., HiSeqX (Illumina) and DNBSEQ-G400 (MGI) for three and one individual(s) of *P. pellucida*, respectively. Full sequences of mitochondrial DNA were constructed by mapping short reads to that of *P. multitentaculata* (LC503532) as a reference using CLC Genomics Workbench ver. 8.0.1 (Qiagen). Gene annotation including rRNA estimation and tRNA prediction was carried out using Geneious Prime Java ver. 11.0.14.1 + 1 (Biomatters Ltd., Auckland, New Zealand) and MITOS2 (Donath et al. 2019) according to Yonezawa et al. (2020). The complete mitochondrial DNA sequences in this study were submitted to the DDBJ/EMBL/GenBank databases with accession numbers LC785386–LC785391, respectively.

Comparison of gene arrangement in the complete mitochondrial DNA sequence was manually performed using schematic diagrams based on annotated sequences. The phylogenetic tree was constructed by means of the maximum likelihood method using MEGA X (Kumar et al. 2018). Nucleotide sequences of complete mitochondrial DNA for the following species were also obtained from the databases: *Prosthiostomum siphunculus* (KT363736), *Enchiridium* sp. (KT363734), *Miroplana shenzhensis* (NC_062124), *Dugesia japonica* (AB618487), *Girardia* sp. (KP090061), *Diversibipalium* sp. (MZ561470), and *Bipalium adventitium* (MZ561467). *Echinococcus canadensis* (AB208063) was used as the outgroup species. Phylogenetic trees were also constructed using partial mitochondrial DNA sequences including cytochrome *b* and cytochrome *c* oxidase subunit I (COI) genes.

## Results

### Phylogenetic Relationship of Planocerid Flatworms

Complete mitochondrial DNA sequences obtained from *Planocera* sp. and *P. pellucida* demonstrated that the gene arrangement was different between toxic and non-toxic planocerids (Fig. S1). The tRNA-Gln gene was arranged between NADH dehydrogenase subunit 5 (ND5) and tRNA-Ser genes in TTX-bearing planocerid species including *P. multitentaculata* and *P. reticulata*, whereas it was detected in the position between tRNA-Leu and tRNA-Lys genes in *P. pellucida*, which correspond to positions seen in other non-toxic flatworms.

**Fig. 3:**
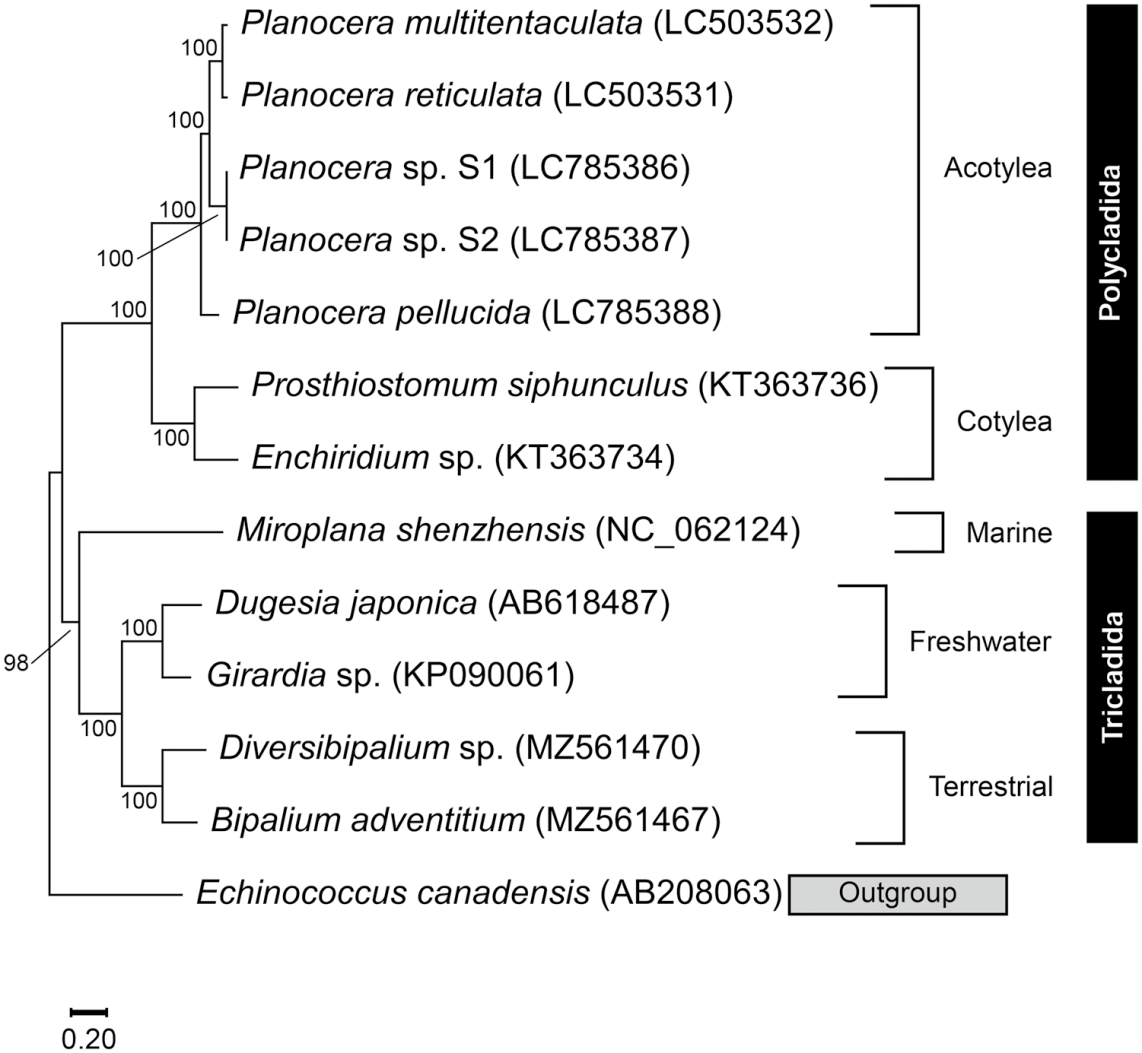
Maximum likelihood tree of the planocerid flatworms and related species in Platyhelminthes inferred from an alignment of complete mitochondrial DNA sequences. Numbers at branches denote the bootstrap percentages from 1000 replicates. The accession numbers LC785386–LC785388 shown in parentheses have been deposited in the DDBJ/EMBL/GenBank databases. *Echinococcus canadensis* was used as the outgroup. Only bootstrap values exceeding 50% are presented. The scale refers to nucleotide substitutions per site

Phylogenetic analysis was performed using these complete mitochondrial DNA sequences, along with previously reported flatworm sequences. The maximum likelihood tree showed that *Planocera* sp. and *P. pellucida* clustered with other planocerid flatworm species, *P. multitentaculata* and *P. reticulata*, with high bootstrap support (Fig. 3). However, the TTX-bearing flatworm *Planocera* sp. clustered with *P. multitentaculata* and *P. reticulata*, diverging at the root with the non-toxic *P. pellucida*. The phylogenetic tree based on sequences of cytochrome *b* and COI genes also showed that within Acotylea, non-toxic and toxic planocerid species diverged and *Planocera* sp. formed a cluster with toxic species (Fig. S2).

### Composition of TTX and Analogs from Four Planocerid Flatworms

**Fig. 4:**
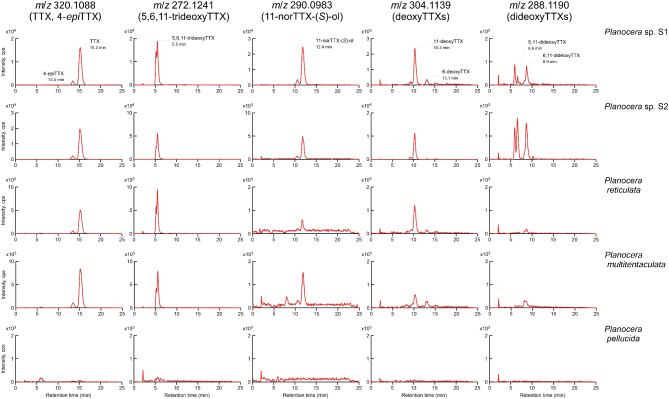
Comparison of extracted ion chromatograms for tetrodotoxin and its analogs from *Planocera* sp. (S1 and S2), *Planocera reticulata*, *Planocera multitentaculata*, and *Planocera pellucida*. Panels for *m*/*z* 320.1088: TTX and 4-*epi*TTX; *m*/*z* 272.1241: 5,6,11-trideoxyTTX; *m*/*z* 290.0983: 11-norTTX-6(*S*)-ol; *m*/*z* 304.1139: deoxyTTXs; *m*/*z* 288.1190: dideoxyTTXs. cps, counts per second

As shown in Fig. 4, peaks for TTX and several compounds related to TTX were commonly detected by LC–MS analysis in two individuals of *Planocera* sp. corresponding to those seen in *P. multitentaculata*. The peak at 15.2 min in *m*/*z* 320.1088 was identical to TTX with 4-*epi*TTX, one of the chemically equilibrium molecules of TTX, at 13.5 min. The peak at 5.5 min in *m*/*z* 272.1241 was identical to 5,6,11-trideoxyTTX, while that at 12.4 min in *m*/*z* 290.0983 was identical to 11-norTTX-6(*S*)-ol. In addition, peaks at 10.3 and 13.1 min in *m*/*z* 304.1139 were identical to 11-deoxyTTX and 6-deoxyTTX, respectively. Peaks at 6–9 min in *m*/*z* 288.1190 would be considered the ions identical to 5,11-dideoxyTTX and 6,11-dideoxyTTX. Similar patterns for TTX and related compounds were also observed in *P. reticulata*. On the other hand, no peaks for TTX and related compounds were detected in *P. pellucida*.

### Effects of Toxification with *Planocera multitentaculata* Egg Plates on Composition of TTX and Analogs in Predatory Fish

**Fig. 5:**
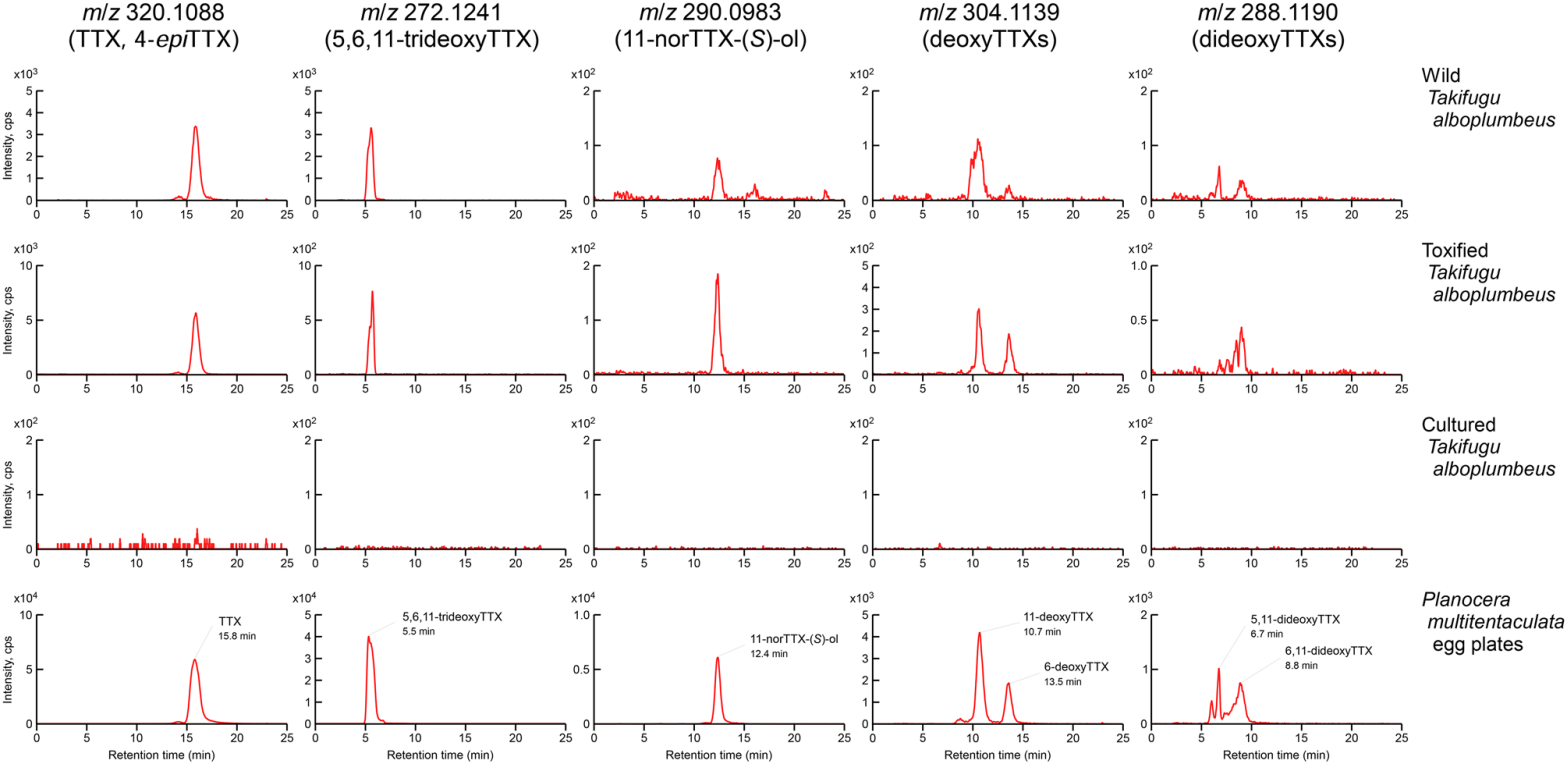
Effects of toxification using the toxic flatworm *Planocera multitentaculata* egg plate on the composition of tetrodotoxin (TTX) in the pufferfish *Takifugu alboplumbeus*. Extracted ion chromatograms represent TTX and its analogs from wild, toxified, and cultured *T. alboplumbeus* individuals. Wild pufferfish juveniles were collected from Okinawa Island, Japan (C in Fig. 1), whereas non-toxic fish juveniles were raised with commercial feed pellets after artificial fertilization at Enoshima, Japan (D in Fig. 1). Culture pufferfish juveniles were toxified by feeding on the flatworm *P. multitentaculata* egg plates. Panels for *m*/*z* 320.1088: TTX and 4-*epi*TTX; *m*/*z* 272.1241: 5,6,11-trideoxyTTX; *m*/*z* 290.0983: 11-norTTX-6(*S*)-ol; *m*/*z* 304.1139: deoxyTTXs; *m*/*z* 288.1190: dideoxyTTXs. cps, counts per second

**Fig. 6:**
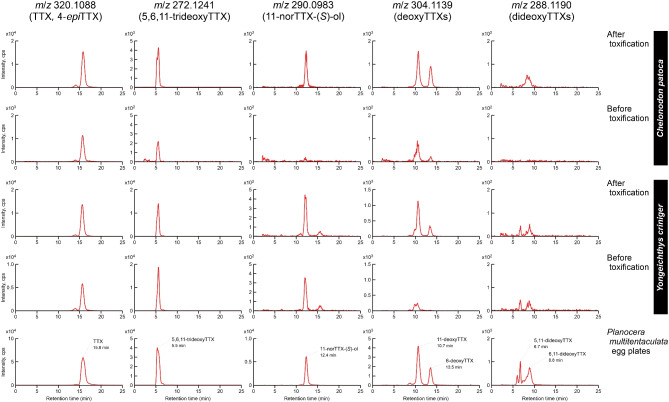
Changes in the composition of tetrodotoxin (TTX) and its analogs in TTX-bearing fish juveniles in association with feeding on the toxic flatworm *Planocera multitentaculata* egg plates. Extracted ion chromatograms represent TTX and analogs from the pufferfish *Chelonodon patoca* (after/before toxification), toxic goby *Yongeichthys criniger* (after/before toxification), and egg plates of the flatworm *Planocera multitentaculata*. Juvenile fish collected at Okinawa Island (C in Fig. 1) were used for toxification experiments after rearing for 1 month with non-toxic commercial pellets. Panels for *m*/*z* 320.1088: TTX and 4-*epi*TTX; *m*/*z* 272.1241: 5,6,11-trideoxyTTX; *m*/*z* 290.0983: 11-norTTX-6(*S*)-ol; *m*/*z* 304.1139: deoxyTTXs; *m*/*z* 288.1190: dideoxyTTXs. cps, counts per second

Composition of TTX and analogs in *Planocera* sp. was identical to that of *P. multitentaculata*, as described above. In addition, the composition of TTX and analogs in adult *P. multitentaculata* individuals was qualitatively similar to those of *P. multitentaculata* egg plates (Fig. S3), as reported in our previous study (Suo et al. 2022). Therefore, toxification experiments were conducted using egg plates of *P. multitentaculata* as the prey for predatory pufferfish and goby. The toxification experiment using the cultured pufferfish *T. alboplumbeus* juveniles demonstrated that the composition of TTX and analogs in artificially toxified juveniles was nearly identical to that of wild juveniles collected at Okinawa Island (Fig. 5). In addition, feeding on *P. multitentaculata* egg plates after rearing with a non-toxic diet for 1 month enhanced the signal intensities of all TTX-related substances in *C. patoca* juveniles, but only deoxyTTXs in *Y. criniger* juveniles (Fig. 6).

**Fig. 7:**
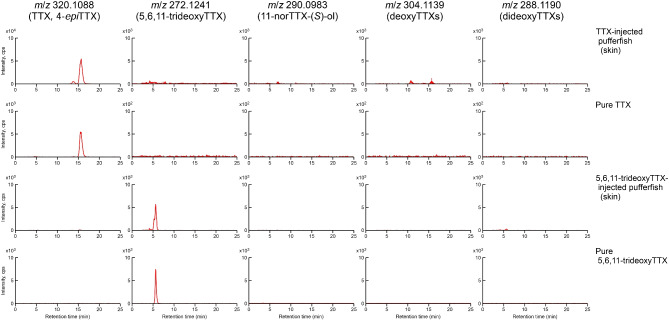
Extracted ion chromatograms for TTX and its analogs in extractions from the pufferfish *Takifugu alboplumbeus* juveniles injected muscularly with TTX and 5,6,11-trideoxyTTX. Extracted ion chromatograms represent TTX and analogs from the TTX-injected pufferfish (skin), pure TTX used injection, 5,6,11-trideoxyTTX-injected pufferfish, and pure 5,6,11-trideoxyTTX used injection (skin). Panels for *m*/*z* 320.1088: TTX and 4-*epi*TTX; *m*/*z* 272.1241: 5,6,11-trideoxyTTX; *m*/*z* 290.0983: 11-norTTX-6(*S*)-ol; *m*/*z* 304.1139: deoxyTTXs; *m*/*z* 288.1190: dideoxyTTXs. cps, counts per second

A single component of TTX and 5,6,11-trideoxyTTX was detected in cultured *T. alboplumbeus* juveniles intramuscularly injected with TTX and 5,6,11-trideoxyTTX, respectively (Fig. 7).

## Discussion

Extensive amounts of TTX have been thought to accumulate in the body of pufferfish via the food chain with bacteria as primary producers (Noguchi et al. 2006; Noguchi and Arakawa 2008), but many missing links among organisms leading to pufferfish have been unexplored. Recently, it has been shown that *T. alboplumbeus* efficiently ingests TTX from *P. multitentaculata* throughout their life history (Itoi et al. 2018; Okabe et al. 2019). It is known that *P. multitentaculata* possesses large amounts of TTX (Miyazawa et al. 1986; Yamada et al. 2017; Itoi et al. 2020b), and with the amount of TTX in the body dependent on body size (Yamada et al. 2017; Itoi et al. 2020b). A similar trend was observed in *P. reticulata*, although data are limited (Itoi et al. 2020b). Planocerid and closely related flatworms, mainly *P. multitentaculata*, have been reported to supply TTX to TTX-bearing marine organisms: *Stylochoplana* sp. to sea slug *P. maculata* (Salvitti et al. 2015); *P. multitentaculata* or closely related flatworms to *C. patoca* and *Y. criniger* (Itoi et al. 2020a); and *P. multitentaculata* to the akazara scallop *Azumapecten farreri akazara* (Okabe et al. 2021) and pufferfish of the genus *Takifugu* (Itoi et al. 2018; Okabe et al. 2019; Ito et al. 2023). However, these manifested relationships would be just the tip of the iceberg, as there are few examples of studies focusing on flatworms.

In the present study, it was shown that compositions of TTX and analogs in *Planocera* sp. and *P. reticulata* were identical to those seen in *P. multitentaculata* (Suo et al. 2022, 2024). On the other hand, none of the TTX and analogs were detected in *P. pellucida*, which is closely related to these toxic flatworms but is not known to possess TTX (Kashitani et al. 2020). Comparison of mitochondrial gene arrangement demonstrated a difference between toxic and non-toxic groups. Phylogenetic analysis based on mitochondrial DNA sequences showed that these TTX-bearing flatworms in the genus *Planocera* form a cluster diverged from non-toxic planocerids at the root, suggesting that these toxic planocerids may possess the gene clusters necessary for the possession/accumulation of TTX (Kashitani et al. 2020), and these toxic species may be potential TTX sources in marine environments. Furthermore, since TTX was also detected in several flatworm species, including marine and terrestrial flatworms (Tanu et al. 2004; Stokes et al. 2014; Salvitti et al. 2015; Kashitani et al. 2020; Suo et al. 2021), the possibility that these toxic flatworms share a common symbiotic bacterial flora cannot be ruled out. Further research progress on remaining questions is expected in the future.

On the other hand, it is known that the components of TTX analogs are different between marine and terrestrial TTX-bearing organisms, suggesting that the biosynthetic pathways of TTX are different in both environments (Kudo et al. 2016; Ueyama et al. 2018; Sato et al. 2021). These implicate the possibility of inferring prey species comparing compositions of TTX and analogs. In the waters around the Ryukyu Islands, DNA fragments of *P. multitentaculata* have been detected in the intestinal contents of TTX-bearing fish, *C. patoca* and *Y. criniger*, and in the water itself at their collection site (Itoi et al. 2020a; Ito et al. 2022). In the present study, a toxification experiment, wherein cultured *T. alboplumbeus* were fed with *P. multitentaculata* eggs, also reproduced the composition pattern of TTX and analogs in wild *T. alboplumbeus* juveniles collected from the Ryukyu Islands. *Takifugu alboplumbeus* is widely distributed in the Japanese Archipelago including the Ryukyu Islands (Takagi et al. 2019), and can be made nontoxic in cultured individuals (Matsui et al. 1982), making it an appropriate model for toxification with TTX. The experiment for intramuscular administration of TTX and its analogs in *T. alboplumbeus* demonstrated that transformation among TTX and its analogs were not observed (Kono et al. 2008). Similar experiments in this study showed that transformation between TTX and 5,6,11-trideoxyTTX did not occur in the body of *T. alboplumbeus*. Since *Planocera* sp. has a similar TTX and analog composition to those of *P. multitentaculata* and *P. reticulata*, it is highly likely that *Planocera* sp. is also involved in the toxification of TTX-bearing fish in the Ryukyu Islands.

The present study on toxification using TTX-bearing planocerid egg plates leaves unanswered why the signal intensities of TTX and analogs, especially 5,6,11-trideoxyTTX, were significantly enhanced in *C. patoca*, but not in *Y. criniger*, contrary to prior expectations. It has been reported that pufferfish juveniles accumulated 2- to fivefold more TTX than 5,6,11-trideoxyTTX, regardless of collection site or species, and that the TTX/5,6,11-trideoxyTTX ratios in pufferfish juveniles were reproduced in the toxification experiment using the *P. multitentaculata* egg plates (Ito et al. 2023). Further, the ratio of TTX to 5,6,11-trideoxyTTX varied among *Y. criniger* populations collected from various sampling locations (Ito et al. 2022). Although it was thought that the difference in composition of TTX and analogs between pufferfish and toxic goby might be the result of different compositions in the prey species and/or differing uptake capacity of TTX and analogs between the two fish groups (Ito et al. 2022). This study could not reach a conclusion on this issue.

However, all of the TTX-bearing planocerids, in this study, had similar compositions of TTX and analogs, leaving the possibility that *Y. criniger* accumulates TTX and analogs by preying on TTX-bearing organisms other than toxic flatworms. For example, ribbonworms including *Cephalothrix simula*, which possess large amounts of TTX (Miyazawa et al. 1988; Asakawa et al. 2000, 2013; Vlasenko et al. 2018; Malykin et al. 2021), might be potential TTX sources for TTX-bearing fish, because *C.* cf. *simula* possesses TTX throughout its life history including planktonic larval stages (Malykin et al. 2023). In the future, we plan to examine the intestinal contents of various TTX-bearing organisms to clarify the missing link between TTX-bearing prey and predators.

### Supplementary Information

Below is the link to the electronic supplementary material.

Supplementary file1 (PDF 310 KB)

## Data Availability

No datasets were generated or analysed during the current study.
