# Supplementary material for: Japanese Planocerid Flatworms: Difference in Composition of Tetrodotoxin and Its Analogs and the Effects of Ingestion by Toxin-Bearing Fishes in the Ryukyu Islands, Japan
Source: Mar Biotechnol (NY). 2024 Apr 17;26(3):500–10. doi: 10.1007/s10126-024-10312-0 (PMC11178581; doi:10.1007/s10126-024-10312-0)
Supplement: Supplementary file 1 — Supplementary file1 (PDF 310 KB) [file 10126_2024_10312_MOESM1_ESM.pdf]

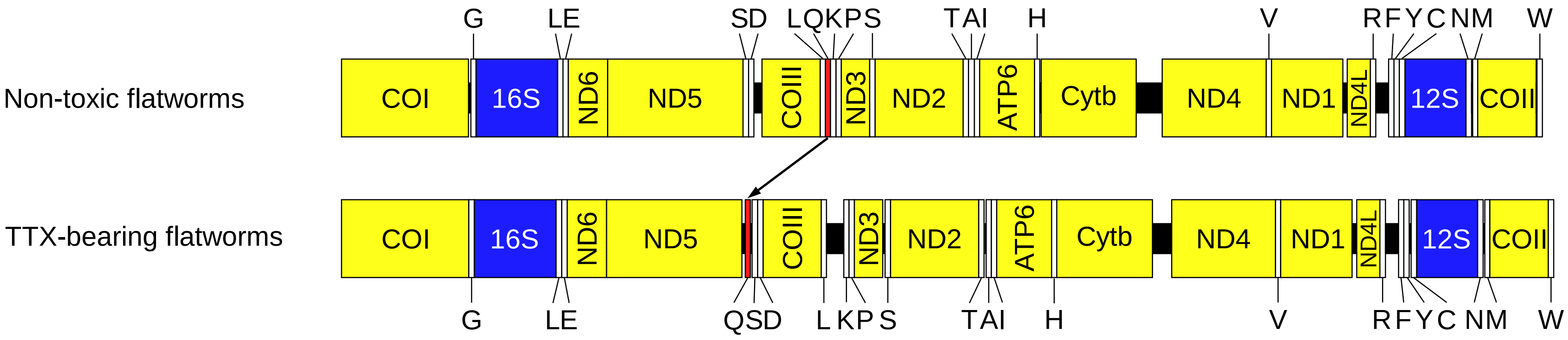

Fig. S1. Comparison of schematic gene arrangement in the complete mitochondrial DNA from non-toxic flatworms with that from TTX-bearing flatworms. The schematics of non-toxic and TTX-bearing flatworms were based on the sequences of *Planocera pellucida* and *Planocera* sp., respectively. Transfer RNA (tRNA), ribosomal RNA (rRNA) and protein-coding genes are annotated with open, blue and yellow boxes, respectively, whereas only a rearranged gene, tRNA-Gln, is represented by a red box. 16S and 12S indicate the genes encoding 16S and 12S rRNAs, respectively; COI - III encode subunits of cytochrome *c* oxidase; ND1 - 6/4L encode subunits of NADH dehydrogenase; Cytb encodes cytochrome *b*; ATPase 6 encode a subunit of F_oF_1-ATP synthase. tRNA-coding genes are represented by their single-letter amino acid codes.

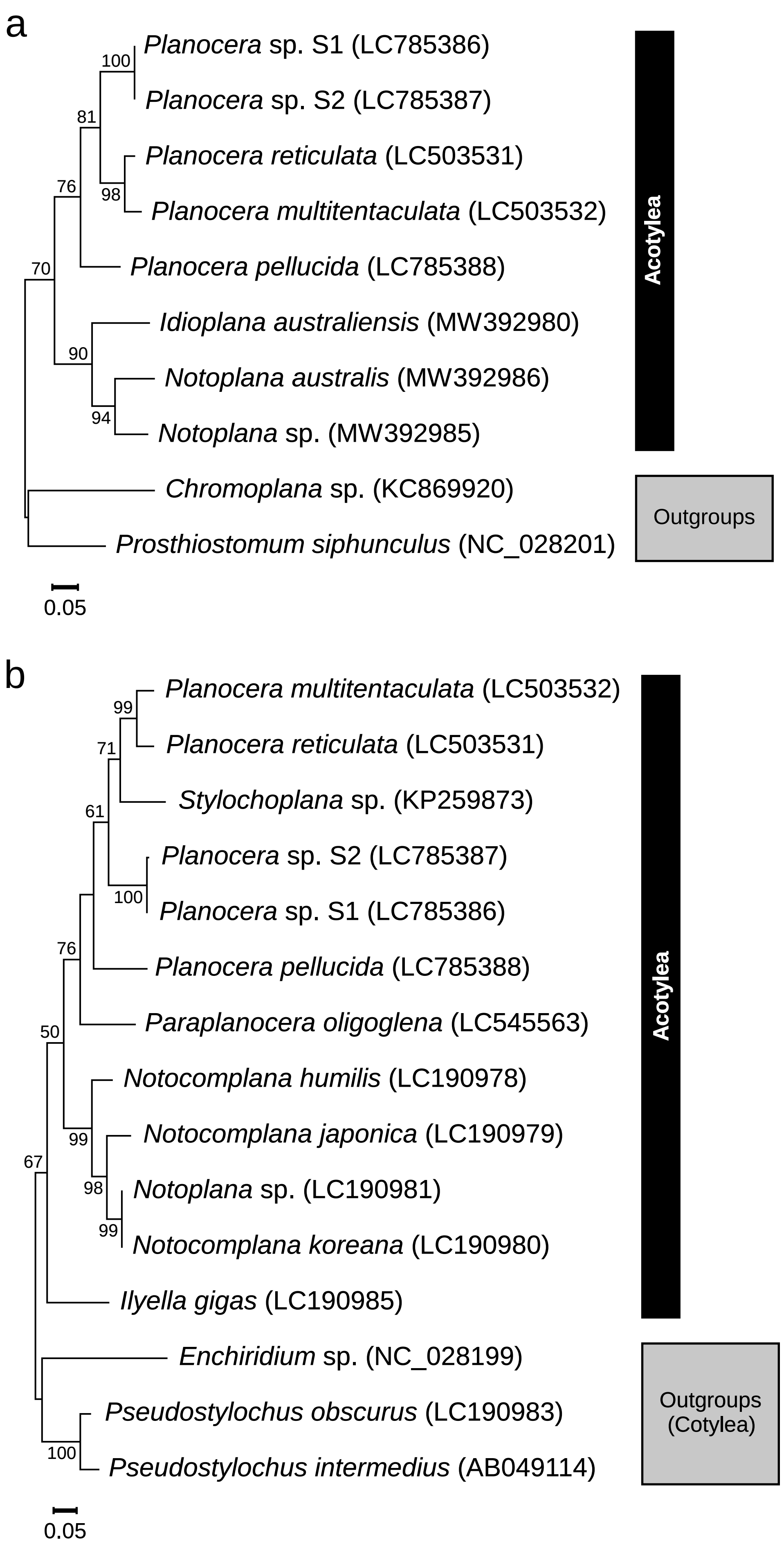

Fig. S2. Maximum likelihood tree of the planocerid flatworms and related species inferred from an alignment of sequences cytochrome *b* gene (**a**) and partial cytochrome *c* oxidase subunit I gene (**b**). Numbers at branches denote the bootstrap percentages from 1000 replicates. The accession numbers LC785386 - LC785388 shown in parentheses have been deposited in the DDBJ/EMBL/GenBank databases. Only bootstrap values exceeding 50% are presented. The scale refers to nucleotide substitutions per site.

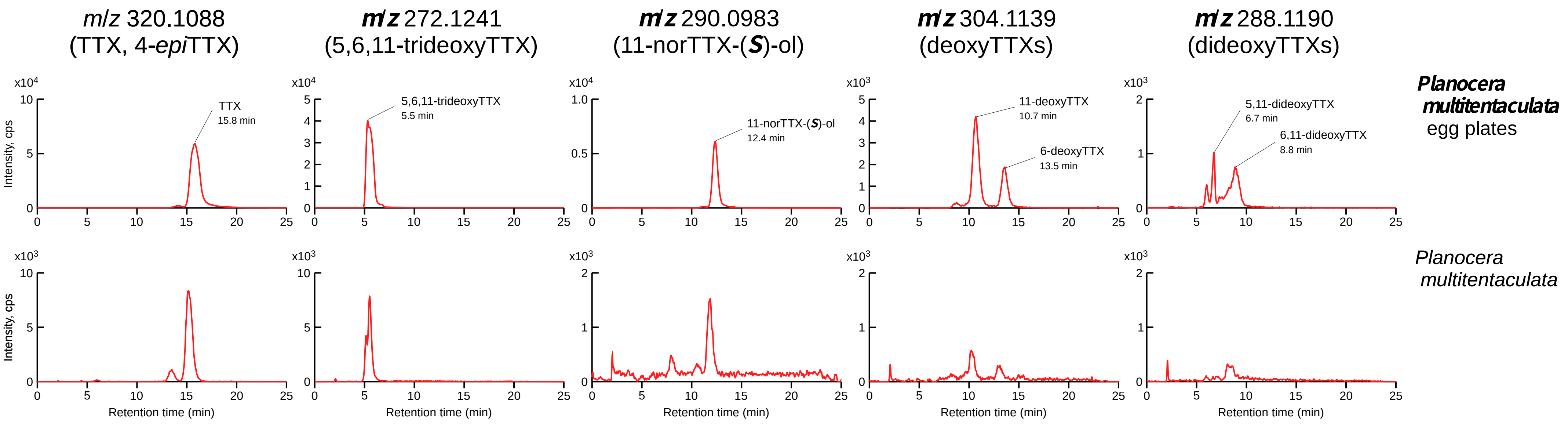

Fig. S3. Comparison of extracted ion chromatograms for tetrodotoxin and its analogs in extractions from egg plate and adult specimen of *Planocera multitentaculata*. Panels for *m*/*z* 320.1088: TTX and 4-*epi*TTX; *m*/*z* 272.1241: 5,6,11-trideoxyTTX; *m*/*z* 290.0983: 11-norTTX-6(*S*)-ol; *m*/*z* 304.1139: deoxyTTXs; *m*/*z* 288.1190: dideoxyTTXs. cps = counts per second.
